# Ischemic postconditioning inhibits apoptosis in an *in vitro* proximal tubular cell model

**DOI:** 10.3892/mmr.2015.3344

**Published:** 2015-02-12

**Authors:** XIAODONG WENG, LEI WANG, HUI CHEN, XIUHENG LIU, TAO QIU, ZHIYUAN CHEN

**Affiliations:** Department of Urology, Renmin Hospital of Wuhan University, Wuhan University, Wuhan, Hubei 430060, P.R. China

**Keywords:** kidney, ischemic postconditioning, *in vitro* model, apoptosis, caspase cascade

## Abstract

Ischemia-reperfusion is a common injury of clinical ischemic disease and surgical lesions. Ischemic postconditioning (IPO) improves the ability of organs subjected to ischemia to tolerate injury. However, renal IPO studies have been based on animal models. In order to gain insights into IPO-induced alterations at the cellular level, an *in vitro* model for IPO was designed using the rat proximal tubular cell line NRK-52E. This model was established by placing NRK-52E cells in ischemic conditions for 3 h, then exposing cells to three cycles of reperfusion for 10 min and finally to ischemic conditions for 10 min (postconditioning). The cells were cultured further in reperfusion conditions for 3, 6, 12 and 24 h. Flow cytometry and Hoechst were used to assess apoptosis. The protein expression of B-cell lymphoma 2 (Bcl-2), Bcl-2-associated X protein (Bax), caspase-3, cleaved caspase-3 and caspase-8 were analyzed by western blotting. The results demonstrated that apoptosis occurred in cells subjected to ischemia/reperfusion (I/R) alone or with postconditioning following reperfusion for 24 h. Cells subjected to I/R demonstrated increased expression of Bax, cleaved caspase-3 and caspase-8 at the end of reperfusion. However, the levels of Bax, cleaved caspase-3 and caspase-8 were significantly attenuated in cells, which had undergone IPO. In conclusion, apoptosis was observed in cells subjected to 3 h of ischemia-reperfusion injury and IPO was able to inhibit this apoptosis. IPO inhibited apoptosis by inhibiting the caspase pathway thereby exerting protective effects.

## Introduction

One of the major causes of acute renal failure (ARF) is ischemia, which occurs in kidney transplantation, partial nephrectomy, renal artery angioplasty, sepsis, accidental or iatrogenic trauma, hydronephrosis, elective urological operations, aortic bypass surgery, cardiopulmonary bypass, the use of vasoconstricting drugs and certain hypotensive states (1,2). ARF has a high incidence in intensive care units, representing an isolated prognostic factor in patients with multiple organ dysfunction syndrome (3). The clinical significance of ARF is due to its high mortality, which ranges between 30 and 70% (4). Thus, novel therapies are required to prevent or alleviate ischemic injury.

Previous studies have demonstrated that ischemic preconditioning (IPR) and ischemic postconditioning (IPO) are two important mechanical methods, which are able to improve the ability of organs subjected to ischemia to tolerate injury (5,6). Although IPR is effective at reducing ischemia-reperfusion injury (IRI), its clinical application is limited as it must be initiated prior to the ischemic period, which is unreasonable in a clinical situation. IPO is a series of brief rapid intermittent cycles of ischemia applied at the onset of reperfusion in the previously ischemic tissue or organ (7). Several studies have demonstrated that IPO was able to cause a significant reduction in the systemic inflammatory response, inhibit the expression of apoptosis-associated molecules and activate endogenous protective molecules (8–10). In renal IPO studies, major studies were based on animal models, including our earlier studies using rat or canine models (11,12). However, to the best of our knowledge, an *in vitro* postconditioning model, which is able to effectively simulate the process of IPO against IRI in the kidney, has not yet been investigated. Based on a study using an *in vitro* model for 13), a novel IPO model, which simulates IPO in the kidney was developed in the present study using a rat proximal tubular cell line (NRK-52E cells). In addition, the molecular mechanism involved in *in vitro* IPO of renal tubular epithelial cells was analyzed.

## Materials and methods

### Cell culture

The renal tubular epithelial cell line, NRK-52E, was purchased from the Cell Resource Center of the Shanghai Institutes for Biological Sciences, Chinese Academy of Sciences (Shanghai, China). The cells were cultured on culture dishes with 5% CO_2_ and maintained at pH 7.4 and 37°C. The medium was changed once every 3 days and the cells were used for experiments at day 10 after seeding. Cells were cultured in serum-free medium for 24 h prior to the experiments. Cells were seeded on 6-well plates or culture dishes as appropriate.

### In vitro IPO model

**Figure 1:**
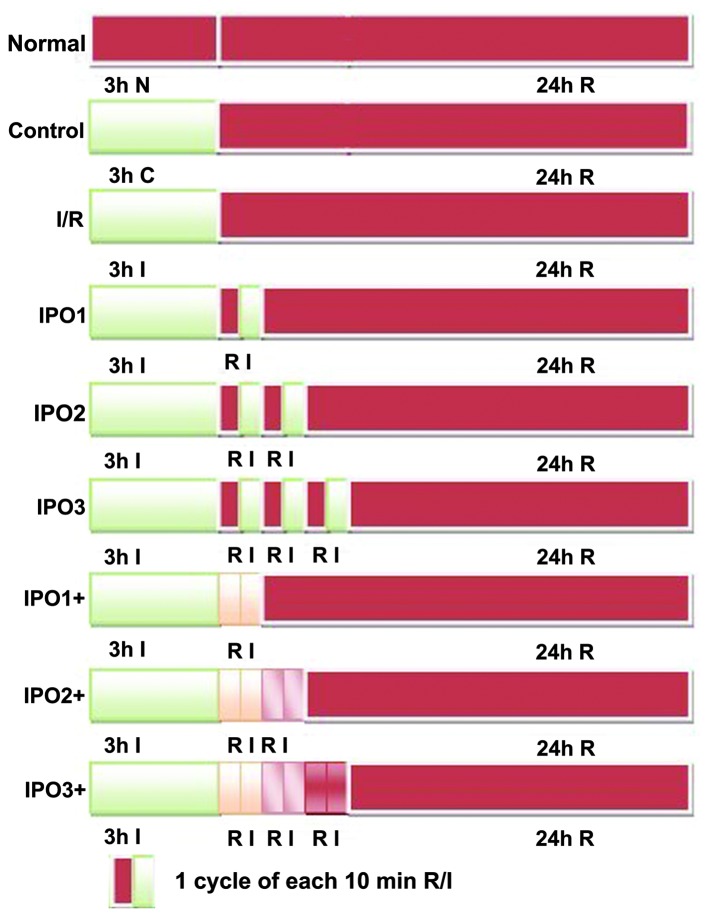
Experimental procedure used to determine the effect of IPO following I/R in an *in vitro* model. Normal, normal condition culture; control, cells cultured in control medium followed by reperfusion; I/R, cells cultured in ischemic conditions followed by reperfusion; IPO, cells cultured in ischemic conditions for 3 h, then replaced with complete medium and cultured under normal conditions for 10 min, followed by placing cells in ischemic conditions for 10 min. IPO1 group, one cycle of IPO; IPO2, two cycles of IPO; IPO3, three cycles of IPO. IPO+, cells cultured under mimic ischemic conditions for 3 h. The ischemic buffer was not changed and another 0.5 ml fresh complete medium was added. The cells were grown under normal conditions for 10 min. Following this, the cells were exposed to ischemic conditions for 10 min without changing the mixed medium. IPO1+, one cycle of IPO+; IPO2+, two cycles of IPO+; IPO3+, three cycles of IPO+. I, ischemia; R, reperfusion; IPO, ischemic postconditioning.

Prior to the experiment, the cells were placed in serum-free medium for 24 h. Subsequently, all cell culture dishes were randomly divided into nine groups (Fig. 1). For the normal group, the cells were cultured in complete medium under normal conditions (5% CO_2_, saturated humidity and 37°C) and 3 h later fresh medium was added and cultured under the same conditions for 24 h. For the control group, the cells were cultured in control buffer(NaHCO_3_ 24.0 mM, Na_2_HPO_4_ 0.8 mM, NaH_2_PO_4_ 0.2 mM, NaCl 86.5 mM, KCl 5.4 mM, CaCl_2_ 1.2 mM, MgCl_2_ 0.8 mM, HEPES 20 mM and 5 mM glucose; pH adjustment to 7.4 with 1 N NaOH) (13) for 3 h and further cultured in complete medium for 24 h. The cells in the ischemia/reperfusion (I/R) group were washed with phosphate-buffered saline (PBS; Gibco Life Technologies, Carlsbad, CA, USA) and placed in ischemic buffer (NaHCO_3_ 4.5 mM, Na_2_HPO_4_ 0.8 mM, NaH_2_PO_4_ 0.2 mM, NaCl 106.0 mM, KCl 5.4 mM, CaCl_2_ 1.2 mM, MgCl_2_ 0.8 mM and morpholinoethanesulfonic acid 20 mM; pH 6.6) (13), and exposed to ischemic conditions (5% CO_2_, 0.5% O_2_, saturated humidity and 37°C) using a tri-gas incubator for 3 h. Subsequently, the cells were placed into the complete medium under normal conditions representing the reperfusion period for 24 h. With respect to the group simulating IPO, there were two approaches: i) Cells were placed in the ischemic buffer and cultured under hypoxic conditions for 3 h and cultured further in complete medium under normal conditions for 10 min. This was followed by placing cells into ischemic buffer and then growing them in ischemic conditions for 10 min. Thus, cells that had undergone one cycle of IPO were termed the IPO1 group. Cells undergoing two or three cycles were termed the IPO2 group and IPO3 group, respectively. Following this, cells were cultured in complete medium under normal conditions for 24 h. ii) Cells were placed in ischemic buffer and cultured under mimic ischemic conditions for 3 h. Subsequently, the complete medium was added and the cells were grown under normal conditions for 10 min. The medium was not replaced with buffer and was directly exposed to ischemic conditions for 10 min. Cells undergoing one cycle were termed the IPO1+ group. According to this, cells undergoing two or three cycles were termed the IPO2+ group and IPO3+ group, respectively. Cells were further placed in complete medium and cultured under normal conditions for 24 h.

### Analysis of apoptosis by flow cytometry

For analysis of apoptosis, 5×10^5^ NRK-52E cells were cultured on a 6-well plate containing 2 ml serum-free medium. After 24 h, all cells were processed in accordance with the above grouping. NRK-52E cells were collected 24 h post-treatment and stained with *Analysis of apoptosis by flow cytometry* (FITC)-conjugated annexin V and propidium iodide (PI) according to the manufacturer’s instructions of the apoptosis detection kit (Annexin V/PI Apoptosis kit; Liankebio, Hangzhou, China). Flow cytometry was performed for analysis of apoptosis (FACSAria; BD Biosciences, Heidelberg, Germany).

### Hoechst 33258 staining

In order to distinguish apoptotic cells from necrotic cells, the cells were stained with Hoechst 33258. The cells from different groups were fixed using Carnoy’s fixative (Beyotime Institute of Biotechnology, Haimen, China) for 10 min. Cells were washed with PBS (pH 7.4), stained with Hoechst 33258 (10 *μ*g/ml) for 5 min at room temperature and microscopically examined (BX-53F; Olympus Corporation, Tokyo, Japan).

### Western blot analysis

The protein expression levels of B-cell lymphoma 2 (Bcl-2), Bcl-2-associated X protein (Bax) caspase-3, caspase-8 and cleaved caspase-3 were examined by western blotting. Briefly, proteins were extracted from NRK-52E cells, separated on 10% SDS-PAGE gels and transferred onto a nitrocellulose membrane (Novex, San Diego, CA, USA). The membranes were blocked with 5% non-fat milk in Tris-buffered saline (Boster Biological Technology, Ltd., Wuhan, China) and Tween 20 buffer (Bio-Rad Laboratories, Inc., Hercules, CA, USA) and incubated with the following rabbit primary antibodies: Bax (1:1,000; cat. no. 2772, Cell Signaling Technology, Inc., Danvers, MA, USA), Bcl-2 (1:1,000; cat. no. 3498, Cell Signaling Technology, Inc.), caspase-3 (1:1,000; cat. no. 9662, Cell Signaling Technology, Inc.), cleaved caspase-8 (1:1,000; cat. no. 9429, Cell Signaling Technology, Inc.) and cleaved-caspase 3 (1:1,000; cat. no. 9661, Cell Signaling Technology, Inc.). All of the primary antibodies were polyclonal, except for the antibody targeting Bcl-2, which was monoclonal. Subsequently, the membranes were incubated with secondary horseradish peroxidase-conjugated goat anti-rabbit IgG antibody (1:2,000; ZDR-5306; ZSGB-BIO, Beijing, China). Specific bands were developed and visualized using an enhanced chemiluminescence detection kit (Immobilon Western Chemiluminescent HRP Substrate; Merck Millipore, Darmstadt, Germany).

### Statistical analysis

All experiments were repeated in triplicate. Data are presented as the mean ± standard error of the mean. For determining the number of apoptotic cells, the groups were compared using one-way analysis of variance and Student-Newman-Keuls test. P<0.05 was considered to indicate a statistically significant difference. All the statistical tests were performed using GraphPad Prism software version 5.0 (GraphPad Software, Inc., San Diego, CA, USA).

## Results

### Apoptotic level in NRK-52E cells following postconditioning

**Figure 2:**
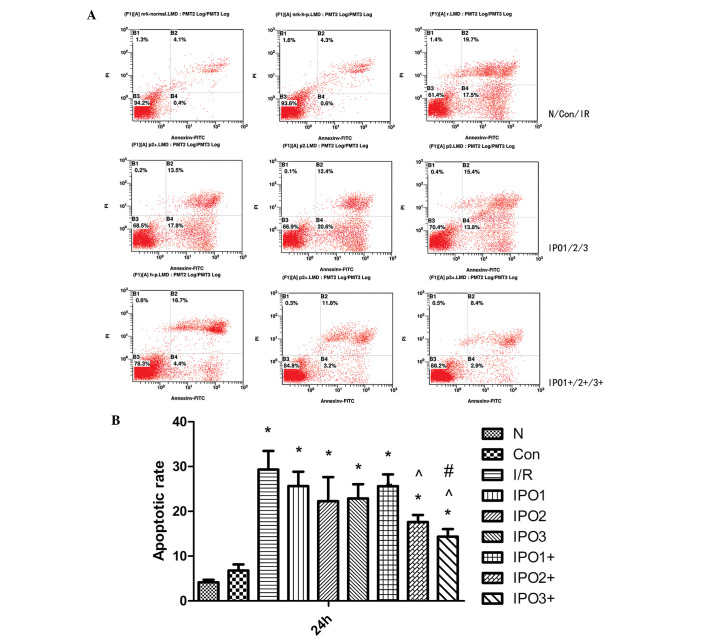
Apoptotic rate of NRK-52E cells following IPO. (A) The apoptotic rate of NRK-52E cells was significantly affected by IPO. The typical flow cytometry results of nine groups cultured for 24 h culture following simulating ischemia and reperfusion injury are shown. (B) Results were obtained from three independent experiments. ^*^P<0.05, versus normal; ^^^P<0.05, versus I/R; ^#^P<0.05, versus IPO1+ (n=3 in each group). IPO, ischemic postconditioning; I/R, ischemia/reperfusion; FITC, fluorescein isothiocyanate.

NRK-52E cells subjected to serum starvation were treated in accordance with the grouping described earlier. After 24 h of culture, NRK-52E were collected and stained with FITC-conjugated annexin V and propidium iodide for detecting apoptosis. As shown in Fig. 2, the rate of apoptosis in the control group was significantly lower than in the I/R, IPO1, IPO2, IPO3 and IPO1+ groups (P<0.05). Compared with the IPO2+ and IPO3+ group, the rate of apoptosis in the I/R, IPO1 and IPO2 groups was significantly higher (P<0.05). In addition, a significant difference between the IPO3 and IPO3+ group (P<0.05) was observed. As the apoptotic rate of the IPO3+ group was lower than in other postconditioning groups, this indicated that this group was the most effective at reducing IRI. Therefore, in the following experiments, the IPO3+ group was the only post-processing method investigated.

**Figure 3:**
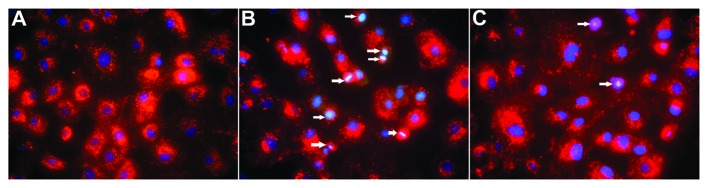
Effect of postconditioning on ischemia and reperfusion-induced apoptosis of NRK-52E cells. (A) Normal, NRK-52E cells cultured under normal conditions. (B) I/R, cells cultured in ischemic conditions and then reperfused for 24 h. (C) IPO3+, cells cultured in ischemic conditions, followed by three cycles of IPO and then reperfused for 24 h. Arrows indicate the apoptotic cells (Hoechst staining; all fluorescence photomicrographs original magnification, ×200).

As shown in Fig. 3, hoechst 33258 staining revealed that the nuclear chromatin was affected. In the control group, faint blue fluorescence was observed in the cell nuclei, which were homogenous. In the I/R group, the blue emission was significantly brighter than in the control group. In I/R cells, condensed chromatin was visible and the formation of apoptotic bodies was observed. Compared with the I/R group, the bright blue emission in the IPO3+ group was clearly attenuated, suggesting that postconditioning was able to ameliorate the injury caused by I/R.

### Expression level of apoptotic proteins in NRK-52E cells due to postconditioning

**Figure 4:**
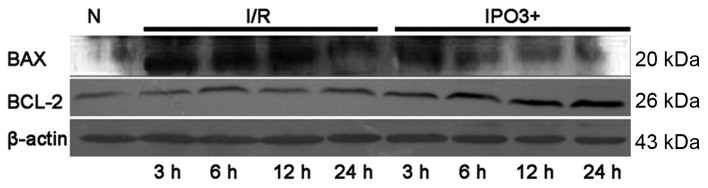
Expression of Bcl-2 and Bax following IPO. The effects of IPO on Bcl-2 and Bax expression in the NRK-52E cells, which were subjected to three cycles of 10 min reperfusion/10 min ischemia for postconditioning. I/R injury increased the expression of Bax and decreased the expression of Bcl-2. However, the alterations induced by I/R injury were opposite in the IPO group. IPO, ischemic postconditioning; Bcl-2, B-cell lymphoma 2; Bax, Bcl-2-associated X protein; I/R, ischemia/reperfusion.

**Figure 5:**
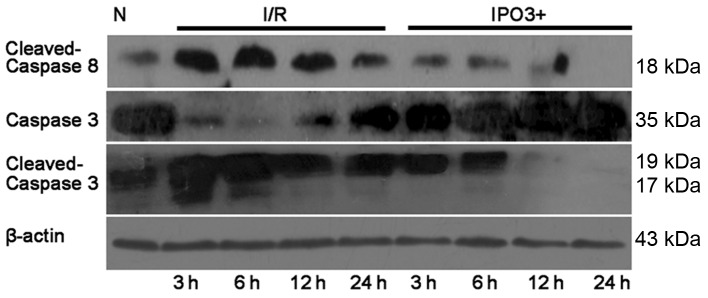
Expression of apoptotic proteins following IPO. The effects of IPO on caspase-3, cleaved caspase-3 and caspase-8 expression in the NRK-52E cells, which were subjected to three cycles of 10 min reperfusion/10 min ischemia for postconditioning. I/R injury increased the expression of cleaved caspase-3 and caspase-8 after 24 h, and decreased the expression of cas-pase-3. However, the alterations induced by I/R injury were opposite in the IPO group. IPO, ischemic postconditioning; I/R, ischemia/reperfusion.

The protein expression levels of Bax, Bcl-2, pro-caspase-3, caspase-8 and cleaved caspase were examined by western blotting (Figs. 4 and 5). The expression of Bcl-2, a crucial inhibitor of the apoptotic process was upregulated in the IPO3+ group, when compared with the control group and I/R group. In addition, with the increase in postconditioning time, the expression of Bcl-2 was increased in the IPO3+ group. Cleaved caspase-3, which is the activated product of pro-caspase-3, has catalytic activity in cell apoptosis. Western blot analysis demonstrated a significant increase in cleaved caspase-3 in the I/R group compared with the control group and with increases in time this trend continued. In the IPO3+ group, the levels of cleaved caspase-3 were clearly restored compared with those in the I/R group. The expression of pro-caspase-3 was decreased in the I/R group and restored in the IPO3+ group. Caspase-8, known to be involved in apoptosis, was significantly increased in the I/R and IPO3+ groups. However, the protein level of caspase-8 was significantly lower in the IPO3+ group compared with the I/R group.

## Discussion

IPO, which was first reported by Zhao, has been demonstrated to be an effective strategy against IRI (14). Previous studies have focused on observing the protective effect of IPO on IRI *in vivo*. However, few studies have focused on this effect in *in vitro* conditions. Although it is not possible to use an *in vitro* model to create an accurate replica of the *in vivo* environment, *in vitro* models have several advantages over *in vivo* as they provide an environment where the specific stimuli can be controlled, isolated and assessed to determine their contribution and effect on physiological or pathophysiological events (15). Thus, in the present study, an *in vitro* postconditioning model was created, which was as close to the *in vivo* IPO pathophysiological environment as possible, using a rat proximal tubular cell line (NRK-52E cells) based on the model used by Sauvant *et al* (13). In this model, all cellular injury caused by reperfusion, including ‘hypoxia, hypercapnia induced acidosis, limited nutrient availability and waste removal impairment’ were taken into account.

Previous studies have established an *in vitro* model to simulate the ischemic environment, one of them using NRK-52E cells with mineral oil overlay (16,17). Although this method was able to approximately establish an *in vitro* model of ischemia, the accuracy of simulation of the ischemic environment was not effective and the protective effect of postconditioning for ischemia was not able to be investigated. A simple model of hypoxia and reoxygenation can be used to simulate cells in IRI (18,19). Nutrients and oxygen deprived/regeneration of the normal culture atmosphere system could simulate ischemia-reperfusion (20,21). However, in the present study, two different methods were used to create an *in vitro* model of ischemia and postconditioning and were compared.

There were two methods to achieve the postconditioning process in the present study. For one approach, following the completion of the pretreatment (24 h serum starvation and 3 h mimic ischemia), 1 ml of complete medium was replaced at each time point during the ischemia-reperfusion cycle. This postconditioning method had certain effects on reducing cell damage and inhibiting cell apoptosis, and compared with the normal group, a significant difference in the apoptotic rate (P<0.05) was observed. For the second approach, following the completion of the pretreatment (24 h serum starvation and 3 h mimic ischemia), the consumed medium was not removed and 0.5 ml fresh medium was added to the dish at each reperfusion cycle. Flow cytometric analysis results indicated that the rate of apoptosis in the IPO+ group was lower than in the IPO group and a significant difference between the IPO3 and IPO3+ group (P<0.05) was observed. This suggested that the IPO+ treatment methods were superior to the IPO approach and the IPO3+ group was selected as the postconditioning group for Hoechst and western blot analysis. The result of Hoechst staining indicated that the IPO3+ group was able to effectively reduce the damage caused by ischemia and reperfusion.

The proteins of the Bcl-2 family, which are crucial regulatory factors, can either promote cell survival, for example Bcl-2, or cell death, for example Bax, by apoptosis (22–24). Increased Bcl-2 can enhance cell survival and evidence indicates that the increased level of Bcl-2 exerts protective effects against apoptosis (25). However, Bax, a key regulator of programmed cell death, is an apoptotic protein and acts by activating caspases (26). The ratio of Bcl-2 to Bax determines the cellular susceptibility to apoptotic stimuli (26–30). The present study found that, as the expression of Bcl-2 gradually increased and Bax gradually decreased, the ratio of the expression of Bcl-2 with Bax was increased in the IPO3+ group compared with the control group and I/R group demonstrating an increased resistance to apoptosis. In addition, with increasing time, this trend became more apparent. It indicated that the postconditioning method in the IPO3+ group was able to inhibit the apoptotic process in NRK-52E cells subjected to simulative ischemia.

Several studies have demonstrated that the caspase family is able to promote and implement cell apoptosis in mammalian cells and caspase-3 is the most crucial downstream apoptotic protease in the caspase cascade (31,32). A number of extracellular signals activate caspase-8 through the Fas receptor pathway and the activation of caspase-8 then promotes caspase-3 activation, which hydrolyzes cell-specific proteins, and poly-ADP ribose polymerase, thus inducing apoptosis (33,34). Pro-caspase-3 itself does not have catalytic activity and it divides into two fragments in the activation process to produce the active form of caspase-3. In the present study, the expression of caspase-3 and caspase-8 was examined by western blot analysis. The result of cleaved caspase-3 and caspase-8 demonstrated a significant decrease in the IPO3+ group compared with the I/R group. In addition, with increasing time this trend became more apparent. In the IPO3+ group subjected to 24 h of culture following postconditioning, the expression of cleaved caspase-3 and caspase-8 decreased. This indicated that this postconditioning method was able to inhibit the activation of the caspase pathway in NRK-52E cells subjected to simulated ischemia.

In *in vivo* conditions, the process of IPO involves repeated renal artery occlusion and opening. The metabolism of the ischemic organ and the nutrient supply are slowly restored to pre-ischemic levels, which are the essence of IPO. In *ex vivo* conditions in the present study, 0.5 ml fresh complete medium was added into the ischemic buffer three times, which leads to gradual restoration of pre-ischemic levels in the extracellular environments (oxygen, PH, nutrient, waste removal impairment) and thus mimic the process of IPO *in vivo*. However, the duration of protective effects of IPO and the exact number of optimal intervals and cycles remain to be elucidated. In addition, *in vitro* models do not fully represent *in vivo* conditions. Therefore, more studies are required to investigate this further.

In the present study, an *in vitro* postconditioning model was established, which was able to effectively simulate the process of IPO against IRI in the kidney. In this model, cells were effectively protected from IRI by IPO. Furthermore, this protection was achieved by inhibiting caspase activation, thereby reducing cell apoptosis. In conclusion, IPO of NRK-52E cells *in vitro* offers a potentially valuable strategy to investigate the protective mechanism of IPO against IRI.

## References

[1] Yun Y, Duan WG, Chen P (2009). Ischemic postconditioning modified renal oxidative stress and lipid peroxidation caused by ischemic reperfusion injury in rats. Transplant Proc.

[2] Barri YM, Sanchez EQ, Jennings LW (2009). Acute kidney injury following liver transplantation: definition and outcome. Liver Transpl.

[3] Schrier RW, Wang W, Poole B, Mitra A (2004). Acute renal failure: definitions, diagnosis, pathogenesis and therapy. J Clin Invest.

[4] Kellum JA (2008). Acute kidney injury. Crit Care Med.

[5] Chen X, Liu X, Wan X, Wu Y, Chen Y, Cao C (2009). Ischemic preconditioning attenuates renal ischemia-reperfusion injury by inhibiting activation of IKKbeta and inflammatory response. Am J Nephrol.

[6] Serviddio G, Romano AD, Gesualdo L (2008). Postconditioning is an effective strategy to reduce renal ischaemia/reperfusion injury. Nephrol Dial Transplant.

[7] Zhao ZQ, Corvera JS, Halkos ME (2003). Inhibition of myocardial injury by ischemic postconditioning during reperfusion: comparison with ischemic preconditioning. Am J Physiol Heart Circ Physiol.

[8] Xing B, Chen H, Zhang M (2008). Ischemic postconditioning inhibits apoptosis after focal cerebral ischemia/reperfusion injury in the rat. Stroke.

[9] Gyurkovics E, Aranyi P, Stangl R (2011). Postconditioning of the lower limb - protection against the reperfusion syndrome. J Surg Res.

[10] Liu XH, Zhang ZY, Sun S, Wu XD (2008). Ischemic postconditioning protects myocardium from ischemia/reperfusion injury through attenuating endoplasmic reticulum stress. Shock.

[11] Chen H, Xing B, Liu X (2008). Ischemic postconditioning inhibits apoptosis after renal ischemia/reperfusion injury in rat. Transpl Int.

[12] Jiang B, Liu X, Chen H (2010). Ischemic postconditioning attenuates renal ischemic/reperfusion injury in mongrel dogs. Urology.

[13] Sauvant C, Schneider R, Holzinger H, Renker S, Wanner C, Gekle M (2009). Implementation of an in vitro model system for investigation of reperfusion damage after renal ischemia. Cell Physiol Biochem.

[14] Zhao H (2007). The protective effect of ischemic postconditioning against ischemic injury: from the heart to the brain. J Neuroimmune Pharmacol.

[15] Russ AL, Haberstroh KM, Rundell AE (2007). Experimental strategies to improve in vitro models of renal ischemia. Exp Mol Pathol.

[16] Witzgall R (1999). The proximal tubule phenotype and its disruption in acute renal failure and polycystic kidney disease. Exp Nephrol.

[17] Meldrum K, Meldrum DR, Hile KL, Burnett AL, Harken AH (2001). A novel model of ischemia in renal tubular cells which closely parallels in vivo inury. J Surg Res.

[18] Cavdar Z, Oktay G, Egrilmez MY (2010). In vitro reoxygenation following hypoxia increases MMP-2 and TIMP-2 secretion by human umbilical vein endothelial cells. Acta Biochim Pol.

[19] Sáenz-Morales D, Escribese MM, Stamatakis K (2006). Requirements for proximal tubule epithelial cell detachment in response to ischemia: role of oxidative stress. Exp Cell Res.

[20] Sáenz-Morales D, Conde E, Escribese MM (2009). ERK1/2 mediates cytoskeleton and focal adhesion impairment in proximal epithelial cells after renal ischemia. Cell Physiol Biochem.

[21] Basnakian AG, Ueda N, Hong X, Galitovsky VE, Yin X, Shah SV (2005). Ceramide synthase is essential for endonuclease-mediated death of renal tubular epithelial cells induced by hypoxia-reoxygenation. Am J Physiol Renal Physiol.

[22] Adams JM, Cory S (1998). The Bcl-2 protein family: arbiters of cell survival. Science.

[23] Ness JM, Harvey CA, Strasser A, Bouillet P, Klocke BJ, Roth KA (2006). Selective involvement of BH3-only Bcl-2 family members Bim and Bad in neonatal hypoxia-ischemia. Brain Res.

[24] Gibson ME, Han BH, Choi J (2001). BAX contributes to apoptotic-like death following neonatal hypoxia-ischemia: evidence for distinct apoptosis pathways. Mol Med.

[25] Martinou JC, Frankowski H, Missotten M, Martinou I, Potier L, Dubois-Dauphin M (1994). Bcl-2 and neuronal selection during development of the nervous system. J Physiol Paris.

[26] Golstein P (1997). Controlling cell death. Science.

[27] Gillardon F, Lenz C, Waschke KF (1996). Altered expression of Bcl-2, Bcl-X, Bax and c-Fos colocalizes with DNA fragmentation and ischemic cell damage following middle cerebral artery occlusion in rats. Brain Res. Mol Brain Res.

[28] Krajewski S, Mai JK, Krajewska M, Sikorska M, Mossakowski MJ, Reed JC (1995). Upregulation of bax protein levels in neurons following cerebral ischemia. J Neurosci.

[29] Gillardon F, Wickert H, Zimmermann M (1995). Up-regulation of bax and down-regulation of bcl-2 is associated with kainate-induced apoptosis in mouse brain. Neurosci Lett.

[30] Oltvai ZN, Milliman CL, Korsmeyer SJ (1993). Bcl-2 heterodimerizes in vivo with a conserved homolog, Bax, that accelerates programmed cell death. Cell.

[31] Prabhakar G, Vona-Davis L, Murray D, Lakhani P, Murray G (2003). Phosphocreatine restores high-energy phosphates in ischemic myocardium: implication for off-pump cardiac revascularization. J Am Coll Surg.

[32] Qi L, Pan H, Li D, Fang F, Chen D, Sun H (2011). Luteolin improves contractile function and attenuates apoptosis following ischemia-reperfusion in adult rat cardiomyocytes. Eur J Pharmacol.

[33] Cho BB, Toledo-Pereyra LH (2008). Caspase-independent programmed cell death following ischemic stroke. J Invest Surg.

[34] Broughton BR, Reutens DC, Sobey CG (2009). Apoptotic mechanisms after cerebral ischemia. Stroke.

